# Therapeutic Targeting of MERTK and BCL-2 in T-Cell and Early T-Precursor Acute Lymphoblastic Leukemia

**DOI:** 10.3390/cancers14246142

**Published:** 2022-12-13

**Authors:** Ryan J. Summers, Juhi Jain, Eleana Vasileiadi, Brittany Smith, Madison L. Chimenti, Tsz Y. Yeung, James Kelvin, Xiaodong Wang, Stephen V. Frye, H. Shelton Earp, Jeffrey W. Tyner, Erik C. Dreaden, Deborah DeRyckere, Douglas K. Graham

**Affiliations:** 1 Aflac Cancer & Blood Disorders Center, Children’s Healthcare of Atlanta and Emory University, Atlanta, GA 30322, USA; 2 Department of Pediatrics, Emory University School of Medicine, Atlanta, GA 30322, USA; 3 Department of Pediatrics, University of Arizona School of Medicine, Tucson, AZ 85724, USA; 4 Department of Pediatrics, Children’s Hospital of Philadelphia, Philadelphia, PA 19104, USA; 5 School of Medicine, Case Western Reserve University, Cleveland, OH 44106, USA; 6 Wallace H. Coulter Department of Biomedical Engineering, Georgia Institute of Technology and Emory University, Atlanta, GA 30332, USA; 7 Center for Integrative Chemical Biology and Drug Discovery, UNC Eshelman School of Pharmacy, Chapel Hill, NC 27599, USA; 8 UNC Lineberger Comprehensive Cancer Center, University of North Carolina Chapel Hill, Chapel Hill, NC 27599, USA; 9 Knight Cancer Institute, Oregon Health Sciences University, Portland, OR 97201, USA

**Keywords:** ETP-ALL, small molecule inhibitor, MERTK, BCL-2, MRX-2843

## Abstract

**Simple Summary:**

Children with T-cell leukemia (T-ALL) who experience relapse have a low chance of a cure with current therapy; so, new medicines are needed. MERTK and BCL-2 are proteins that may be therapeutic targets in children with T-ALL. In this research we tested whether treatments targeting MERTK and BCL-2 are effective in experimental models of T-ALL. We found that MERTK and BCL-2 are present in some T-ALL cells and showed that a new drug called MRX-2843, which blocks MERTK function, can kill T-ALL cells. In mice with T-ALL, treatment with MRX-2843 reduced the presence of leukemia cells and prolonged survival. We also found that MRX-2843 provided more effective T-ALL cell killing when it was combined with another drug called venetoclax, which blocks BCL-2 function. These studies provide good evidence that MRX-2843 could be effective for treatment of T-ALL, especially when combined with venetoclax.

**Abstract:**

T-cell acute lymphoblastic leukemia (T-ALL) accounts for 15% of childhood ALL. The early T-precursor (ETP-ALL) subset is characterized by an immature T-cell phenotype, chemoresistance, and high rates of induction failure. MERTK receptor tyrosine kinase is ectopically expressed in half of T-ALLs, particularly those with an immature T-cell phenotype, suggesting a role in ETP-ALL. The anti-apoptotic protein B-cell lymphoma-2 (BCL-2) is essential for ETP-ALL cell survival. Here, we show that MERTK and BCL-2 mRNA and protein are preferentially expressed in ETP-ALL patient samples. The dual MERTK/FLT3 inhibitor MRX-2843 decreased MERTK activation and downstream signaling, inhibited cell expansion, and induced cell death in ETP-ALL cell lines. Further, 54% (21/39) of primary T-ALL patient samples were sensitive to MERTK inhibition. Treatment with MRX-2843 significantly reduced leukemia burden and prolonged survival in cell-line-derived T-ALL and ETP-ALL xenograft models. In a patient-derived ETP-ALL xenograft model, treatment with MRX-2843 markedly reduced peripheral blood leukemia and spleen weight compared to vehicle-treated mice and prolonged survival. MRX-2843 also synergized with venetoclax to provide enhanced anti-leukemia activity in ETP-ALL cell cultures, with a dose ratio of 1:20 MRX-2843:venetoclax providing optimal synergy. These data demonstrate the therapeutic potential of MRX-2843 in patients with T-ALL and provide rationale for clinical development. MRX-2843 monotherapy is currently being tested in patients with relapsed leukemia (NCT04872478). Further, our data indicate that combined MERTK and BCL-2 inhibition may be particularly effective for treatment of ETP-ALL.

## 1. Introduction

Acute lymphoblastic leukemia (ALL) is the most common form of childhood cancer. T-cell ALL (T-ALL) accounts for approximately 15% of ALL cases in children [1]. Historically, event-free and overall survival rates for patients with T-ALL have been inferior to those with B-lineage ALL (B-ALL), even in the setting of intensified therapy [2,3,4,5,6]. Although more contemporary treatment regimens have improved T-ALL outcomes [1,2], a subset of patients continue to have a poor prognosis.

Early T-precursor ALL (ETP-ALL) comprises 10–15% of pediatric T-ALL, has stem cell and myeloid features, and is difficult to treat, with high rates of remission failure and relapse in children [3,4,5]. ETP-ALL blasts are defined immunophenotypically by the absence of CD8 and CD1a, weak expression of CD5, and the presence of one or more myeloid or stem cell markers [3]. The term near ETP-ALL (Near-ETP) has been used to describe cases that meet the immunophenotypic criteria for ETP-ALL but express CD5 on >75% of blasts [6]. Most patients with ETP-ALL are stratified to high-risk treatment protocols [7] and receive more intensive cytotoxic chemotherapies with a higher incidence of short- and long-term toxic side effects and greater potential to require a bone marrow transplant. Thus, novel, less-toxic therapies are urgently needed to treat this unique subclass of T-ALL.

MERTK is a member of the TAM (TYRO3, AXL, and MERTK) family of receptor tyrosine kinases. The TAM receptors are ectopically or aberrantly expressed and contribute to oncogenesis in a wide range of human malignancies [8,9,10,11]. Ectopic expression of MERTK was first noted in acute lymphoblastic leukemia (ALL) [12] and is present in 30–50% of pediatric B-ALL [13] and T-ALL [14,15] patient samples. Furthermore, MERTK has been validated in preclinical studies as a therapeutic target in B-ALL and T-ALL [13,14,16]. Importantly, shRNA-mediated inhibition of MERTK in T-ALL cell lines inhibited signaling through the JAK/STAT and MAPK pathways, increased chemosensitivity, and prolonged survival in mice with xenografts [14]. MRX-2843 is an orally available small molecule MERTK and FMS-like tyrosine kinase 3 (FLT3) inhibitor that has potent preclinical activity in B-ALL [16], acute myeloid leukemia [17], and solid tumor models [18] and is currently in phase I clinical trials in patients with solid tumors (NCT03510104 and NCT04762199) and leukemia (NCT04872478 and NCT04946890). Further, a phase I trial combining MRX-2843 with the EGFR inhibitor osimertinib (NCT04762199) for patients with EFGR-mutant non-small-cell lung cancer is currently underway.

Numerous studies have demonstrated the dependence of hematopoietic malignancies, including T-ALL, on the anti-apoptotic protein B-cell lymphoma 2 (BCL-2) and its family members [19,20,21]. BCL-2 protein is overexpressed in ETP-ALL patient samples compared to non-ETP T-ALL [22]. Further, ETP-ALL is preferentially sensitive to BCL-2 inhibition [23,24,25], though recent data have shown that the spleen may represent a sanctuary site for residual disease in an ETP-ALL model treated with the BCL-2 inhibitor venetoclax as monotherapy [26].

In this report, we expand on our prior work identifying MERTK as a therapeutic target in T-ALL [14] and demonstrate that ETP-ALL is particularly sensitive to MERTK inhibition. Furthermore, we show that the small molecule MERTK inhibitor MRX-2843 is active in T-ALL both in vitro and in vivo and synergizes with the BCL-2 inhibitor venetoclax. These data support the clinical development of MRX-2843 and provide a rationale for further studies combining MRX-2843 and venetoclax in T-ALL.

## 2. Materials and Methods

### 2.1. Inhibitors

MRX-2843 and UNC2025 were synthesized as previously described [27]. Venetoclax was purchased from LC Laboratories (Woburn, MA, USA). For in vitro studies, stock solutions were prepared in DMSO (Sigma-Aldrich; St. Louis, MO, USA), and the DMSO vehicle control concentrations were equivalent to the highest dose of test agent for each experiment. For in vivo experiments, MRX-2843 was dissolved in saline and venetoclax was prepared as a homogenous suspension in 10% ethanol (EtOH; Sigma-Aldrich), 30% polyethylene glycol 400 (PEG-400; Sigma-Aldrich), and 60% Phosal-50 (Lipoid LLC; Newark, NJ, USA).

### 2.2. RNA Expression Data

Publicly available RNA expression data were queried for MERTK and BCL-2 expression. Read counts for MERTK and BCL-2 expression were normalized to the mean read count of non-ETP patient samples. Data were derived from the Therapeutically Applicable Research to Generate Effective Treatments (TARGET) initiative, phs000464 (https://ocg.cancer.gov/programs/target (accessed on 23 October 2019). The TARGET data used for this analysis are available at https://portal.gdc.cancer.gov/projects (accessed on 23 October 2019).

### 2.3. Cell Lines and Cell Culture

The cell lines CEM, HPB-ALL, HSB2, Jurkat, MOLT4, Loucy, and PEER were obtained from either the American Type Culture Collection (ATCC; Manassas, VA, USA) or the DSMZ (German Collection of Microorganisms and Cell Culture; Braunschweig, Germany). The Jurkat-luciferase cell line has been previously described [28]. The Loucy-luciferase cell line was generously provided by Dr. Pieter Van Vlierberghe (Center for Medical Sciences, Ghent University, Ghent, Belgium). Short tandem repeat microsatellite loci analysis was used to confirm cell line identities. Cultures were confirmed *Mycoplasma*-free. Frozen cell line stocks were generated at the time of authentication and thawed for experiments ≤ 3 months before use. All cell lines were cultured in RPMI medium (Gibco, ThermoFisher Scientific, Waltham, MA, USA) supplemented with 10–20% fetal bovine serum (FBS) and 1% penicillin–streptomycin (complete RPMI (cRPMI)).

### 2.4. Patient Samples

Patient samples (blood or bone marrow) were obtained from the Aflac Leukemia and Lymphoma Biorepository at Children’s Healthcare of Atlanta (samples designated CHOA), Children’s Oncology Group (ETP-0068TJ, ETP-0065FN), and the Therapeutic Advances in Childhood Leukemia & Lymphoma Consortium (TACL0274). Other investigators may have received specimens from the same subjects. Samples ETP-0068TJ and ETP-0065FN were injected into immune-compromised mice and serially passaged to generate murine xenograft models as described below. Additional xenograft-passaged patient samples (ETP1, ETP5, ETP8, ETP12, ETP14, and PATRAP) were generously provided by Dr. David Teachey (Children’s Hospital of Philadelphia, University of Pennsylvania School of Medicine, Philadelphia, PA, USA).

### 2.5. Immunoblot Analysis

Cell lysates were prepared as previously described [17]. Where indicated, cells were treated with vehicle or MRX-2843 for 1 h prior to harvest. For experiments with GAS6 stimulation, cells were spun down and resuspended in 500 μL of GAS6 conditioned medium or unconditioned control medium for 8 min prior to harvest. Proteins were resolved on 8% or 10% Tris-Glycine SDS-PAGE gels (Invitrogen, Waltham, MA, USA) and transferred onto nitrocellulose membranes. Membranes were blocked with 5% milk in tris-buffered saline with 0.1% Tween-20 (TBST) and then incubated with antibodies (Abs) specific to the protein of interest. Proteins were visualized on x-ray film using Western Lightning Plus ECL substrate (Perkin Elmer, Waltham, MA, USA). After detection of phosphorylated proteins, membranes were stripped and probed for total protein or tubulin (loading control). The following Abs were used: MERTK from Abcam no. 52968 (Cambridge, UK), Goat anti-rabbit IgG-HRP from Bio-Rad no. 1706515 (Hercules, CA, USA). BCL-2 (D55G8), α-tubulin (11H10), STAT5 (D3N2B), and phosphorylated STAT5 (Tyr694; D47E7) from Cell Signaling Technology (Danvers, MA, USA). Protein quantitation was performed by densitometry using ImageJ software (NIH; Bethesda, MD, USA).

### 2.6. Immunoblot Analysis of MERTK

Analysis of phosphorylated MERTK was conducted as described in [17]. The following Abs were used: anti-MERTK (MAB8912; R&D Systems; Minneapolis, MN, USA), p-MERTK (Y749, Y753, Y754; Phosphosolutions, Aurora, CO, USA), and MERTK (ab52968; Abcam).

### 2.7. Cell Viability Assays

Cells were plated in triplicate at 20,000 cells/well and cultured in cRPMI. After 24 h, cells were treated with vehicle, MRX-2843, venetoclax, or a combination of MRX-2843 and venetoclax for an additional 48 h and then stained with PrestoBlue™ (Invitrogen) or CellTiter-Glo^®^ (Promega, Madison, WI, USA) cell viability reagents. Fluorescence (PrestoBlue) or luminescence (CellTiter-Glo) was measured using a plate reader. Data shown are representative of at least 3 independent experiments.

### 2.8. Apoptosis Assays

Cells were plated at 300,000 cells/well and cultured in cRPMI for 24 h and treated with vehicle or MRX-2843 for an additional 48 h. Treated cells were then harvested and resuspended in PBS with 1 μM PO-PRO™-1 iodide (Invitrogen) and 1.5 μM propidium iodide (Invitrogen) for 15–30 min prior to assessment of dye uptake by flow cytometry using a Cytoflex flow cytometer (Beckman Coulter, Brea, CA, USA) and FlowJo v10.8.0 analysis software (Becton Dickinson, Franklin Lakes, NJ, USA).

### 2.9. Patient Sample Sensitivity Screening

Blood and bone marrow samples were obtained after receiving informed consent with IRB approval at Oregon Health & Science University (Portland, OR, USA), Stanford University (Palo Alto, CA, USA), University of Utah (Salt Lake City, UT, USA), University of Texas Southwestern (Dallas, TX, USA), and University of Colorado-Denver (Aurora, CO, USA). Mononuclear cells were cultured for 72 h in 384-well plates with graded concentrations of UNC2025 or vehicle, and relative numbers of viable cells were determined as previously described [29].

### 2.10. Murine Xenograft Models

NOD.Cg-*Prkdc^scid^Il2rg^tm1Wjl^/*SzJ (NSG) and NOD.Cg-*Prkdc^scid^Il2rg^tm1Wjl^*Tg (CMV-IL3,CSF2,KITLG)1Eav/MloySzJ (NSGS) mice were purchased from The Jackson Laboratory (Bar Harbor, ME, USA) or bred in-house. Established leukemia cell lines or a xenograft-passaged patient sample were suspended in PBS and injected into the tail vein in NSG or NSGS mice (1 × 10^6^ cells/mouse), respectively, to establish xenografts. Male and female mice were equally represented in all experiments. Lymphoblasts were detected in peripheral blood, spleen, and bone marrow samples after staining with an FITC-conjugated anti-human CD45 Ab (BD Biosciences, East Rutherford, NJ, USA, no. 555482). Samples were analyzed using a Cytoflex flow cytometer (Beckman Coulter) and FlowJo v10.8.0 analysis software (Becton Dickinson). For luciferase-expressing cell lines, bioluminescence images were captured and quantitated at intervals as previously described [28]. The highest measured bioluminescence value was carried forward after mice were removed from the study. Data were collected until <60% of mice remained in that group. Mice with established xenografts were randomized to groups to achieve an equitable distribution of starting disease burden and treated once daily with MRX-2843 or vehicle administered at a volume of 10 mL/kg by oral gavage. Mice receiving treatment were weighed and evaluated daily using a standardized health scoring system and mice with health scores ≥ 13 and/or weight loss >20% were euthanized.

### 2.11. High-Throughput Screening

ETP-ALL cells (Loucy and PEER) were cultured in cRPMI with 10% FBS. Cells were plated (10,000–20,000 cells/well) on white polystyrene 384-well microplates (Greiner Bio-One, Monroe, NC, USA) using a Multidrop Combi Reagent Dispenser (Thermo Fisher, Middlesex County, MA, USA) and then treated with combinations of MRX-2843 (0–800 nM) and venetoclax (0–400 nM) (MedChemExpress, Monmouth Junction, NJ, USA) using high-throughput liquid transfer robotics (Beckman NX Liquid Handler; Beckman Coulter). Prior to liquid transfer, drugs were stored in checkerboard arrays of anhydrous DMSO (Thermo Fisher) on polypropylene microwell plates (VWR, Radnor, PA, USA) such that pairwise drug combinations were tested in quadruplicate at a final DMSO concentration of 0.5% *v*/*v*. Cell plates were incubated for 72 h prior to measuring cell viability with the CellTiter-Glo 2.0 Assay (Promega). Relative growth inhibition was calculated by subtracting background luminescence and normalizing to vehicle controls. Synergy was calculated using a response additivity model with the Explicit Mean Equation, as previously described [30], where values > 1% were categorized as synergistic, values < −1% were antagonistic, and all others were categorized as additive. The Z-factor for each assay was measured >0.5.

### 2.12. Statistics

Data were analyzed by paired or unpaired *t*-test, one-way ANOVA with Dunnett’s test for multiple comparisons, or Mann–Whitney-U test as appropriate and are presented as mean ± SEM. *p* values of <0.05 were considered significant. IC_50_ values were determined by nonlinear best-fit regression analysis with upper and lower values constrained to 1 and 0, respectively. Kaplan–Meier survival curves were compared using the log-rank test. Synergy was calculated using the fractional product method [31] or a response additivity model with the Explicit Mean Equation [30]. All statistical analyses were performed using Prism 9 software, version 9.1.0 (GraphPad Software, San Diego, CA, USA).

### 2.13. Study Approval

All animal experiments were conducted in accordance with the relevant regulatory standards and were approved by the Institutional Animal Care and Use Committee of Emory University.

## 3. Results

### 3.1. MERTK and BCL-2 Are Preferentially Expressed in ETP-ALL

**Figure 1:**
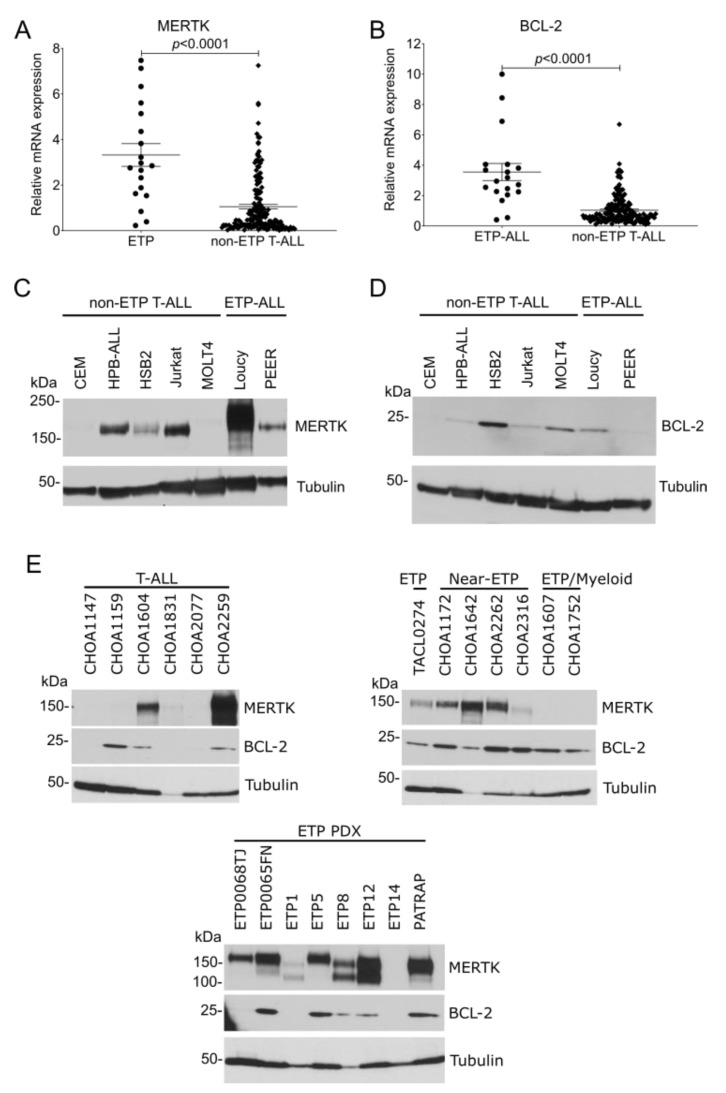
MERTK and BCL-2 mRNA and protein are preferentially expressed in ETP-ALL compared to non-ETP T-ALL. (**A**,**B**) Publicly available gene expression data from the TARGET program were analyzed to determine the relative expression of MERTK (**A**) or BCL-2 (**B**) in pediatric ETP-ALL and non-ETP T-ALL patient samples. mRNA read counts were normalized to the mean mRNA read count in the non-ETP T-ALL samples and the distribution was compared using an unpaired *t*-test. (**C**,**D**) Whole-cell lysates from a panel of T-ALL and ETP-ALL cell lines were probed for MERTK (**C**) or BCL-2 (**D**) expression by immunoblot. Tubulin was used as a loading control. Images shown are representative of at least three independent experiments. (**E**) Whole-cell lysates of primary samples from patients with T-ALL (left panel) or ETP-ALL, near-ETP, and ETP/Myeloid ALL (right panel) or from xenograft-passaged ETP-ALL patient samples (ETP PDX, bottom panel) were probed for MERTK and BCL-2. Tubulin was used as a loading control.

To evaluate MERTK and BCL-2 mRNA expression in patients with ETP-ALL and non-ETP T-ALL, publicly available gene expression data from the TARGET program were analyzed. MERTK (Figure 1A) and BCL-2 (Figure 1B) mRNAs were significantly overexpressed in ETP-ALL samples compared with non-ETP T-ALL samples. We have previously shown that the MERTK protein is ectopically expressed in T-ALL cell lines [14] and pediatric T-ALL patient samples [14,15]. We expanded on that work by comparing MERTK and BCL-2 expression in ETP-ALL cell lines and patient samples with non-ETP T-ALL cell lines and patient samples. MERTK protein was expressed in 2/2 (100%) ETP-ALL cell lines and 3/5 (60%) non-ETP T-ALL cell lines (Figure 1C). BCL-2 protein was expressed in 1/2 (50%) ETP-ALL cell lines and 4/5 (80%) T-ALL cell lines (Figure 1D). Further, MERTK was expressed in 7/8 (87.5%) xenograft-passaged ETP-ALL patient samples and 5/7 (71.4%) ETP, near-ETP, or ETP/myeloid primary patient samples, but in only 3/6 (50%) non-ETP T-ALL primary patient samples (Figure 1E). Similarly, BCL-2 was expressed in 5/8 (62.5%) xenograft-passaged ETP-ALL patient samples and 7/7 (100%) ETP, near-ETP, or ETP/myeloid primary patient samples, but only 3/6 (50%) non-ETP T-ALL primary patient samples (Figure 1F). Thus, MERTK and BCL2 mRNA and protein are preferentially expressed in ETP-ALL relative to other T-ALLs.

### 3.2. MERTK Activation and Downstream Signaling Is Inhibited by MRX-2843 in ETP-ALL Cell Lines

**Figure 2:**
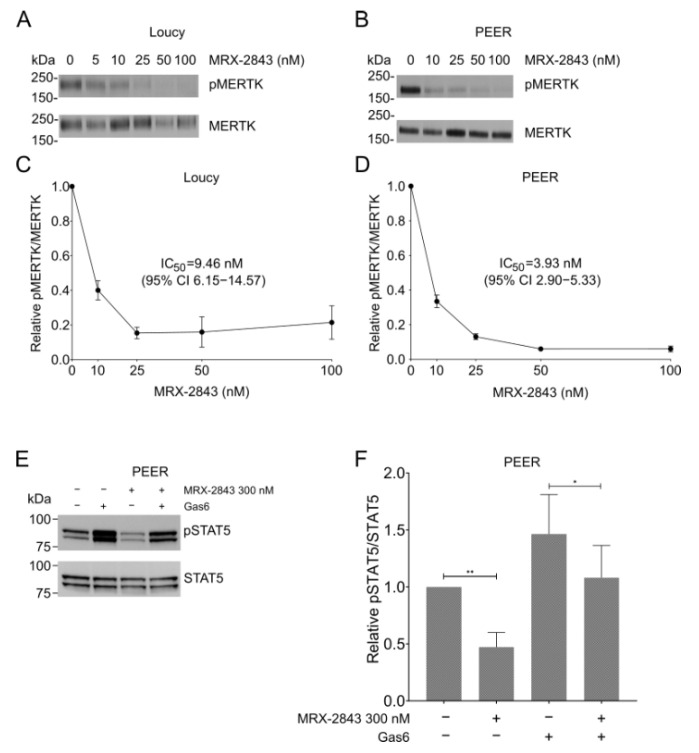
Treatment with MRX-2843 inhibits activation of MERTK in ETP-ALL cell lines in a dose-dependent manner. (**A**–**D**) Loucy (**A**,**C**) and PEER (**B**,**D**) cells were treated with the indicated concentrations of MRX-2843 or vehicle (DMSO). (**A**,**B**) After 1 h, cells were treated with phosphatase inhibitor; then, cell lysates were prepared, MERTK protein was immunoprecipitated, and pMERTK and total MERTK were detected by immunoblot. Images shown are representative of results obtained in three independent experiments. (**C**,**D**) Proteins were quantitated by densitometry and pMERTK/MERTK ratios relative to vehicle control were determined. IC_50_ values were calculated by nonlinear best-fit regression. Mean values and standard errors derived from 3–4 independent experiments are shown. (**E**,**F**) PEER cells were treated with 300 nM MRX-2843 or vehicle with or without Gas6 ligand, and phosphorylated and total STAT5 proteins were detected by immunoblot. Images shown are representative of results obtained in at least three independent experiments. (**F**) Proteins were quantitated by densitometry and pSTAT5/STAT5 ratios were determined relative to vehicle treatment. Mean values and standard errors derived from six independent experiments are shown. * *p* < 0.05 and ** *p* < 0.01 by paired *t*-test.

MERTK was active in both the Loucy (Figure 2A) and PEER (Figure 2B) cell lines, as indicated by detection of phosphorylated MERTK (pMERTK) in the absence of MRX-2843. Furthermore, treatment with MRX-2843 inhibited auto-phosphorylation of MERTK in a dose-dependent manner in both the Loucy (Figure 2A,C) and PEER (Figure 2B,D) cell lines. Decreased phosphorylation was seen at concentrations as low as 10 nM, with near-complete inhibition at 100 nM. MRX-2843 inhibited MERTK activation with IC_50_ values of 9.5 nM (95% confidence interval (CI) 6.2–14.6) in the Loucy cell line (Figure 2C) and 3.9 nM (95% CI 2.9–5.3) in PEER (Figure 2D). Additionally, MERTK inhibition decreased downstream signaling via STAT5 in PEER cells (Figure 2E,F). Treatment with 300 nM MRX-2843 reduced STAT5 activation by 53% (*p* = 0.0093) in the presence of FBS, which contains the MERTK ligands GAS6 and PROS1, and by 38% (*p* = 0.0465) when additional GAS6 was added to the culture to provide an acute stimulus (Figure 2E,F). Similar assays were attempted with Loucy cells but pSTAT5 was not detected (data not shown). Similarly, although we previously demonstrated reduced activation of AKT and ERK in response to MERTK inhibition in non-ETP T-ALL models [14], pAKT and pERK were not phosphorylated in PEER cells, and treatment with MRX-2843 did not affect pAKT and pERK levels in Loucy cells (data not shown).

### 3.3. MRX-2843 Mediates Functional Anti-Leukemia Effects in ETP-ALL Cell Lines

**Figure 3:**
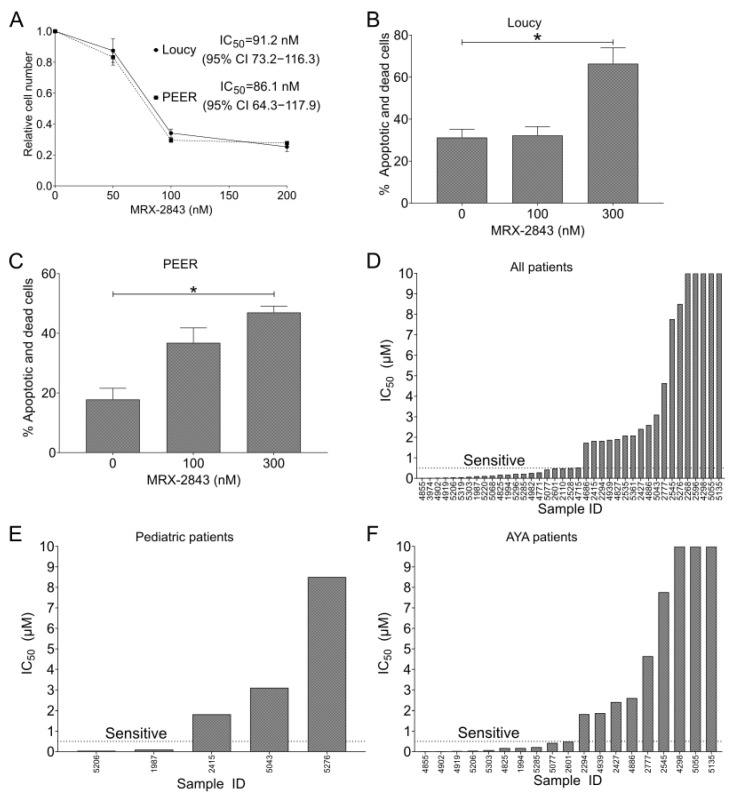
MERTK inhibition has functional antitumor effects in ETP-ALL cell lines and T-ALL patient samples. (**A**) Loucy or PEER cells were plated in triplicate at 20,000 cells/well and cultured for 24 h; then, they were treated with vehicle (DMSO) or MRX-2843 at the indicated concentrations for an additional 48 h. After treatment, cells were stained with Presto Blue reagent, fluorescence was measured, and cell numbers were calculated relative to the DMSO control. IC_50_ values were determined by nonlinear best-fit regression analysis. Mean values ± SEM were derived from three independent experiments. (**B**,**C**) Loucy (**B**) or PEER (**C**) cells were plated at 300,000 cells/well and cultured for 24 h, and then treated with vehicle or MRX-2843 at the indicated concentrations for an additional 48 h. After treatment, cells were stained with PO-PRO-1 iodide and propidium iodide dye uptake was assessed by flow cytometry. Mean values ± SEM were derived from 3–4 independent experiments (* *p* < 0.05, one-way ANOVA). (**D**–**F**) T-ALL patient samples were cultured with UNC2025, a close analogue of MRX-2843, and relative cell numbers were assessed using MTS reagent. (**E**) Patient samples obtained from patients aged 0–18 years. (**F**) Patient samples obtained from patients aged 15–39 years. Abbreviations: AYA—adolescent and young adult.

Treatment with MRX-2843 resulted in a dose-dependent reduction in cell density in both Loucy and PEER cell cultures (Figure 3A). The IC_50_ for MRX-2843 in this assay was 91 nM (95% CI 73–116) in Loucy cultures and 86 nM (95% CI 64–118) in PEER cultures. Furthermore, treatment with MRX-2843 resulted in a significant dose-dependent increase in cell death in Loucy (Figure 3B) and PEER (Figure 3C) cells. In Loucy cultures, treatment with 300 nM MRX-2843 induced cell death in 51% of viable cells (Figure 3B). In PEER cultures, 300 nM MRX-2843 induced cell death in 35% of viable cells (Figure 3C). The same experiment was conducted in Jurkat T-ALL cells and demonstrated a non-significant trend toward increased cell death at a dose of 300 nM MRX-2843 (*p* = 0.055; Figure S1).

### 3.4. Half of Pediatric and Adult T-ALL Patient Samples Are Susceptible to MERTK Inhibition Ex Vivo

A panel of primary T-ALL patient samples was screened for sensitivity to MERTK inhibition using UNC2025 [16], a close analogue of MRX-2843 (Figure 3D–F). Fifty-four percent (21/39) of primary T-ALL patient samples were sensitive to UNC2025 with an IC_50_ ≤ 550 nM (Figure 3D), including 2/5 (40%) pediatric samples (0–18 years; Figure 3E) and 10/19 (53%) adolescent/young adult samples (15–39 years; Figure 3F).

### 3.5. MRX-2843 and Venetoclax Mediate Synergistic Anti-Leukemia Activity in ETP-ALL Cell Lines

**Figure 4:**
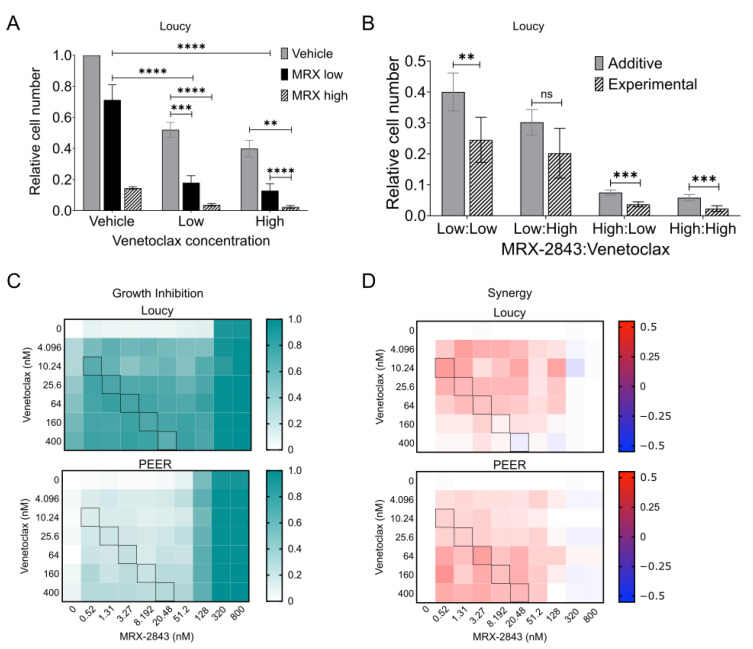
MRX-2843 and venetoclax mediate synergistic anti-leukemia activity in ETP-ALL cell lines. (**A**,**B**) Loucy cells were plated in triplicate at 20,000 cells/well and cultured for 24 h before treatment with vehicle, MRX-2843 (low = 62.5 nM, high = 125 nM), venetoclax (low = 250 nM, high = 500 nM), or MRX-2843 and venetoclax combined for an additional 48 h. After treatment, cells were stained with CellTiter-Glo reagent, luminescence was measured, and cell numbers were calculated relative to the DMSO control. Mean values ± SEM were derived from four independent experiments. For (**B**), expected additive values were calculated using the fractional product method and compared with experimentally observed values (** *p* ≤ 0.01, *** *p* ≤ 0.001, **** *p* ≤ 0.0001, unpaired *t*-test). (**C**,**D**) ETP-ALL cell lines (Loucy and PEER) were treated in quadruplicate with pairwise drug combinations of MRX-2843 and venetoclax, and relative cell numbers were determined utilizing a high-throughput platform and the CellTiter-Glo readout. (**C**) Relative growth inhibition was calculated by subtracting background luminescence and normalizing to vehicle controls. (**D**) Synergy was calculated using the Response Additivity model. Values >1% indicate synergistic, values < −1% are antagonistic, and all others are additive. Black boxes in (**C**,**D**) represent combinations of MRX-2843:venetoclax at a 1:20 dose ratio. Abbreviations: ns – not significant.

Treatment with MRX-2843 or the BCL-2 inhibitor venetoclax significantly reduced relative cell numbers in cultures of the Loucy cell line (Figure 4A and data not shown). As a monotherapy, MRX-2843 mediated a 29% reduction in cell density at a concentration of 62.5 nM and an 85% reduction at a concentration of 125 nM. Venetoclax reduced relative cell numbers by 48% at a dose of 250 nM and by 60% at a dose of 500 nM. At all doses tested, combined treatment with both agents resulted in significant reductions in cell density compared to either single agent. Moreover, the interaction between MRX-2843 and venetoclax was synergistic (Figure 4B). Specifically, treatment with the combination resulted in a statistically significant decrease in cell number compared to the expected value for an additive interaction in cultures treated with low/low, high/low, and high/high doses of MRX-2843/venetoclax and a trend toward decreased cell number in response to the low/high combination. Synergistic interactions are characterized by a ratio between the observed experimental cell density and the expected additive cell density (O/E) that is less than one. O/E ratios were 0.613 (low/low), 0.668 (low/high), 0.493 (high:low), and 0.398 (high:high). To more comprehensively evaluate the combination and identify a dosing strategy to provide optimal synergy, we used a high-throughput approach to screen 70 pairwise dose combinations of MRX-2843 with venetoclax in the ETP-ALL cell lines Loucy and PEER. As expected, growth inhibition increased with increasing concentration of both agents (Figure 4C), although MRX-2843 was again more potent as a monotherapy than venetoclax, with near-complete growth inhibition at a concentration of 320 nM. A molar ratio of 1:20 MRX-2843:venetoclax provided optimal synergy, defined as the ratio that maintained the highest level of synergy at all dose combinations across both ETP-ALL cell lines (Figure 4D).

### 3.6. MRX-2843 Reduces Disease Burden and Prolongs Survival in Orthotopic T-ALL and ETP-ALL Cell Line Xenograft Models

**Figure 5:**
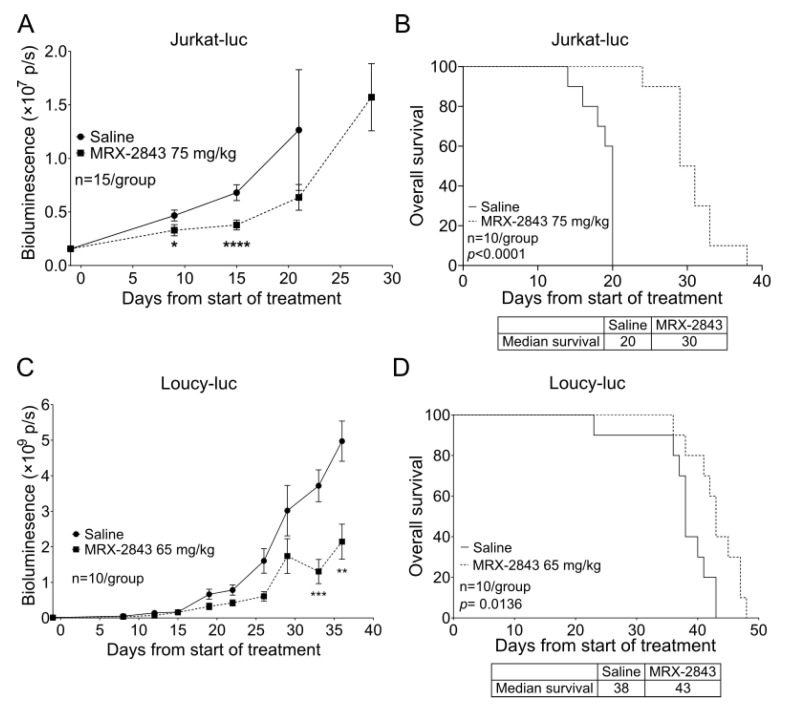
MRX-2843 monotherapy delays disease progression and prolongs survival in orthotopic T-ALL and ETP-ALL xenograft models. (**A**–**D**) NSG mice were injected with a luciferase-expressing T-ALL cell line cells and once-daily oral treatment with MRX-2843 or saline vehicle was initiated after engraftment of disease. (**A**,**C**) Disease burden was monitored at intervals by bioluminescence imaging. (**B**,**D**) Survival was monitored. (**A**,**B**) Mice inoculated with luciferase-expressing Jurkat cells were treated with 75 mg/kg MRX-2843 or saline for 28 days. (**A**) Mean bioluminescence intensities and SEM are shown (* *p* < 0.05, **** *p* < 0.0001, Mann–Whitney-U test, n = 15). (**B**) Survival was significantly prolonged in mice with Jurkat xenografts treated with MRX-2843 (*p* < 0.0001, log-rank test, n =10). (**C**,**D**) Mice inoculated with luciferase-expressing Loucy cells were treated with 65 mg/kg MRX-2843 or saline until removal from the study. (**C**) Mean bioluminescence intensities and SEM are shown (** *p* ≤ 0.01, *** *p* ≤ 0.001, Mann–Whitney-U test, n = 10). (**D**) Survival was significantly prolonged in mice with Loucy xenografts treated with MRX-2843 (*p* = 0.0136, log-rank test, n = 10).

In mice with xenografts of a luciferase expressing Jurkat T-ALL cell line [28], treatment with 75 mg/kg MRX-2843 significantly reduced disease burden, indicated by reduced bioluminescence (Figure 5A), and prolonged survival by 50% compared with vehicle-treated control mice (Figure 5B). Treatment with 65 mg/kg MRX-2843 also significantly reduced disease burden (Figure 5C) and extended median survival from 38 to 43 days in a luciferase-expressing xenograft model of the ETP-ALL cell line Loucy (Figure 5D). A repeat experiment in this model using the same dose of MRX-2843 yielded similar results (Figure S2); although, in this case, the prolongation in median survival seen in mice treated with MRX-2843 did not reach statistical significance.

### 3.7. MRX-2843 Reduces Disease Burden and Prolongs Survival in a Patient-Derived Xenograft Model of ETP-ALL

**Figure 6:**
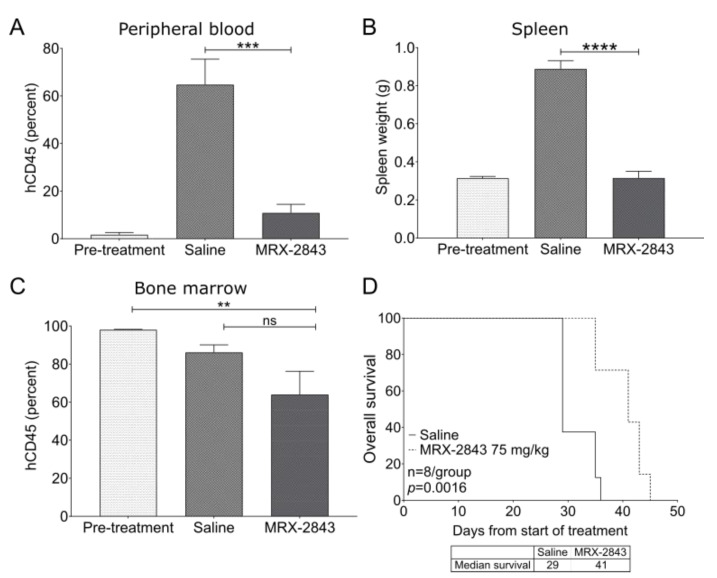
MRX-2843 monotherapy promotes leukemia clearance in peripheral blood and spleen, and prolongs survival in a patient-derived ETP-ALL xenograft model. (**A**–**D**) NSGS mice were inoculated with xenograft-passaged cells from a patient with ETP-ALL (ETP0068TJ) and treatment was initiated 36 days later. (**A**–**D**) A cohort of mice were harvested prior to the start of treatment (pre-treatment). The remaining mice were treated once daily with 75 mg/kg MRX-2843 or saline vehicle. (**A**–**C**) After treatment for 29 days, peripheral blood (**A**), spleen (**B**), and bone marrow (**C**) were collected and leukemic blasts (hCD45+) were detected by flow cytometry. Spleen weight was used as a surrogate for splenic disease burden. Mean values ± SEM are shown (** *p* ≤ 0.01, *** *p* ≤ 0.001, **** *p* ≤ 0.0001, one-way ANOVA, n = 6). (**D**) Survival was monitored in the remaining mice and was significantly prolonged in mice treated with MRX-2843 (*p* = 0.0016, log-rank test, n = 8). Abbreviations: ns—not significant.

Treatment with MRX-2843 significantly reduced peripheral blood disease burden by 84.3% (Figure 6A) and splenic disease burden by 64.5% (Figure 6B) compared with saline-treated controls in the 0068TJ ETP-ALL PDX model. Although mice treated with MRX-2843 had a 34.7% reduction in bone marrow disease burden compared with pre-treatment controls (*p* < 0.01), there was no significant difference between mice treated with MRX-2843 compared with vehicle-treated controls (Figure 6C). However, survival was significantly prolonged from a median of 29 days in vehicle-treated mice to 41 days in mice treated with MRX-2843 (*p* = 0.0016; Figure 6D). A repeat experiment in this model produced similar results (Figure S3).

## 4. Discussion

Despite recent improvements in overall survival for children with T-ALL [1,2], outcomes for children with relapsed disease remain poor [32]. Patients with ETP-ALL have an increased risk of induction failure, which is associated with inferior outcomes [5]. We previously reported that MERTK is ectopically expressed in T-ALL patient samples and cell lines [14,15] and is a potential therapeutic target in T-ALL [14]. In this study, we validated and extended our prior work in T-ALL and demonstrated that MERTK is preferentially expressed in ETP-ALL patient samples compared with non-ETP T-ALL. Similarly, other groups have demonstrated that ETP-ALL is dependent on BCL-2 [23] and that BCL-2 is a therapeutic target in ETP-ALL [23,24,26,33,34,35]; we expanded on those works by demonstrating preferential expression of BCL-2 in ETP-ALL patient samples. These findings suggest that BCL-2-directed therapy may be particularly effective in patients with ETP-ALL.

In this study, we utilized MRX-2843—a dual MERTK/FLT3 inhibitor currently in clinical trials—to probe the anti-leukemia effects of MERTK inhibition in ETP-ALL and T-ALL models. MRX-2843 inhibited MERTK activation, decreased relative cell numbers, and induced cell death in two ETP-ALL cell lines. Additionally, approximately 50% of T-ALL patient samples were sensitive to UNC2025, a nearly identical analogue of MRX-2843 with the same target selectivity [27], in ex vivo assays. Similarly, we previously demonstrated that 50% of T-ALL patient samples express MERTK [14]. This concordance between the fraction of cells expressing MERTK and the fraction that are sensitive to UNC2025 implicates on-target MERTK inhibition as a mechanism of MRX-2843/UNC2025 anti-leukemia activity, although MERTK expression data were not available to directly test the concordance in individual samples.

MRX-2843 inhibited MERTK activation at a lower IC_50_ than was required to mediate functional anti-leukemia effects, though these findings are consistent with what we have previously observed in B-ALL [16] and AML [17]. While we cannot rule out the possibility that some anti-leukemia effects of MRX-2843 are mediated by off-target kinase inhibition, we have evidence to suggest this is not the case. First, the in vivo effects of MRX-2843 in the Jurkat model phenotypically copy the effect of shRNA-mediated knockdown of MERTK that we have previously reported [14]. Further, pharmacologic MERTK inhibition has no effect on the growth of mononuclear cells isolated from healthy donor human cord blood and bone marrow at concentrations ≤ 500 nM [16].

Mutations in FMS-like tyrosine kinase 3 (*FLT3*) have been reported to occur at a higher frequency in patients with ETP-ALL, though the frequency appears to be lower in pediatric patients with ETP-ALL as compared to adults [36,37,38]. Pediatric ETP-ALL patient samples have been shown to preferentially express FLT3 protein compared with non-ETP T-ALL samples [37]; however, it is not clear whether this translates to protein expression. We have shown that MRX-2843 can mediate anti-leukemia effects through inhibition of both MERTK and FLT3 in AML models [17]. We were not able to consistently demonstrate FLT3 expression in our ETP-ALL models (data not shown); therefore, we did not pursue further investigation of this pathway as an alternative target for MRX-2843 in this context.

Importantly, MRX-2843 was therapeutically effective in several murine T-ALL and ETP-ALL xenograft models. With once-daily oral dosing, treatment with MRX-2843 slowed disease progression and prolonged survival in orthotopic MERTK-expressing ETP-ALL and T-ALL cell line and patient-derived xenograft models. These data support the continued clinical development of MRX-2843 for patients with T-ALL, including those with ETP-ALL. Indeed, adolescents and adults with relapsed T-ALL are eligible to receive MRX-2843 in an ongoing clinical trial (NCT04872478).

While MRX-2843 was effective in mice with T-ALL/ETP-ALL, mice treated with the monotherapy were not cured. Thus, we explored the potential to combine MRX-2843 with other therapies to enhance therapeutic efficacy. Indeed, MRX-2843 synergized with the BCL-2 inhibitor venetoclax in two ETP-ALL cell lines and provided increased anti-leukemia activity against ETP-ALL cells compared with either of the drugs alone. Given the biologic rationale for targeting BCL-2 in T-ALL and ETP-ALL, and the robust clinical experience with venetoclax, this combination is particularly promising. Using a high-throughput screen, we also identified an optimal synergistic ratio of 1:20 MRX-2843:venetoclax. These data will inform upcoming in vivo studies combining MRX-2843 and venetoclax in T-ALL and ETP-ALL mouse models. The recent finding that ETP-ALL cells may find sanctuary from venetoclax monotherapy in the spleen [26], coupled with the robust activity of MRX-2843 in the splenic compartment in our study, provides further rationale for this combination.

Although PEER cells did not express detectable BCL-2 protein in our assays, they retained limited sensitivity to BCL-2 monotherapy at high concentrations. This may reflect an off-target effect. Alternatively, BCL-2 protein expression may not correlate with sensitivity to BCL-2 inhibition, a finding that has been demonstrated in T-ALL cell lines [23]. The development of alternative biomarkers of sensitivity to BCL-2 inhibition, such as BH3 profiling, will be important in identifying patients who may benefit most from BCL-2-directed therapy.

Activation of the JAK/STAT signaling pathway—in particular, activation of STAT5—is also important for ETP-ALL cell survival [22]. We previously demonstrated MERTK-mediated activation of JAK/STAT signaling via STAT5 in non-ETP T-ALL [14]. Here, we show that MRX-2843 also abrogates downstream signaling via STAT5 in an ETP-ALL cell line. Given that JAK/STAT inhibition is known to be an effective therapeutic approach in ETP-ALL models [22], combined inhibition of MERTK and JAK/STAT represents another intriguing combination worthy of further investigation.

In this study, we have demonstrated that MERTK inhibition mediates direct anti-leukemia effects in MERTK-expressing T-ALL and ETP-ALL models. Using Mertk −/− mouse models, our group has previously demonstrated that MERTK deficiency also promotes immune-mediated clearance of ALL [39]. Further studies have demonstrated these findings with Mertk −/− mice and MERTK small-molecule inhibitors. Further, the mechanism of this anti-leukemia response was shown to be due, at least in part, to a T-cell/dendritic cell interaction in the bone marrow microenvironment [40]. While beyond the scope of this project, we hypothesize that MERTK plays a similar role in the leukemia microenvironment in T-ALL. Therefore, MERTK serves as a dual therapeutic target in ALL, mediating both direct anti-leukemia effects and anti-leukemia immunity in the bone marrow microenvironment. In this case, the therapeutic potential of MRX-2843 is likely to increase in the context of an intact immune system compared to the studies in immune-compromised mice reported here.

## 5. Conclusions

Our findings support the ongoing clinical development of MRX-2843 for treatment of T-ALL. Additionally, they provide a rationale for further preclinical investigation of combined MERTK and BCL-2 inhibition in ETP-ALL and T-ALL models. Based on these and other data, MRX-2843 monotherapy is being tested in patients with relapsed acute leukemia (NCT04872478).

## Data Availability

The data presented in this study are available on request from the corresponding author.

## Supplementary Materials

The following supporting information can be downloaded at: https://www.mdpi.com/article/10.3390/cancers14246142/s1, Supplementary File S1: Figure S1: MERTK inhibition increases cell death in the T-ALL cell line Jurkat; Figure S2: MRX-2843 monotherapy delays disease progression and prolongs survival in an orthotopic ETP-ALL xenograft model; Figure S3: MRX-2843 monotherapy promotes leukemia clearance in the peripheral blood and spleen, and prolongs survival in a patient-derived xenograft model of ETP-ALL; Supplementary File S2: The original Western Blots include unprocessed Western blot films from Figure 1 and Figure 2.
