# Supplementary material for: Therapeutic Targeting of MERTK and BCL-2 in T-Cell and Early T-Precursor Acute Lymphoblastic Leukemia
Source: Cancers (Basel). 2022 Dec 13;14(24):6142. doi: 10.3390/cancers14246142 (PMC9776749; doi:10.3390/cancers14246142)
Supplement: Supplementary file 1 [file cancers-14-06142-s001.zip › cancers-2019469-supplementary file 1.pdf]

Figure S1

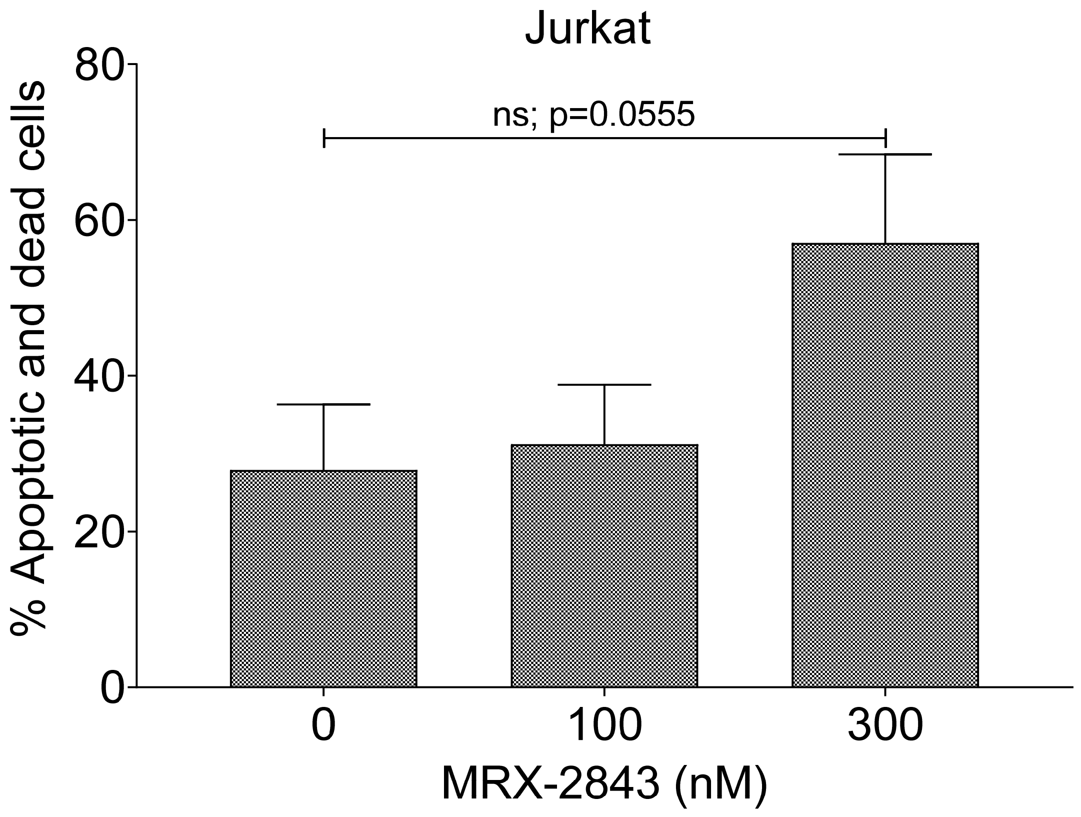

Figure S1. MERTK inhibition increases cell death in the T-ALL cell line Jurkat. Jurkat cells were plated at 300,000 cells/well and cultured for 24 hours, then treated with vehicle or MRX-2843 at the indicated concentrations for an additional 48 hours. After treatment, cells were stained with PO-PRO-1 iodide and propidium iodide dye uptake was assessed by flow cytometry. Mean values ± SEM were derived from 4 independent experiments. p=ns by one-way ANOVA. Abbreviations: ns – not significant

Figure S2

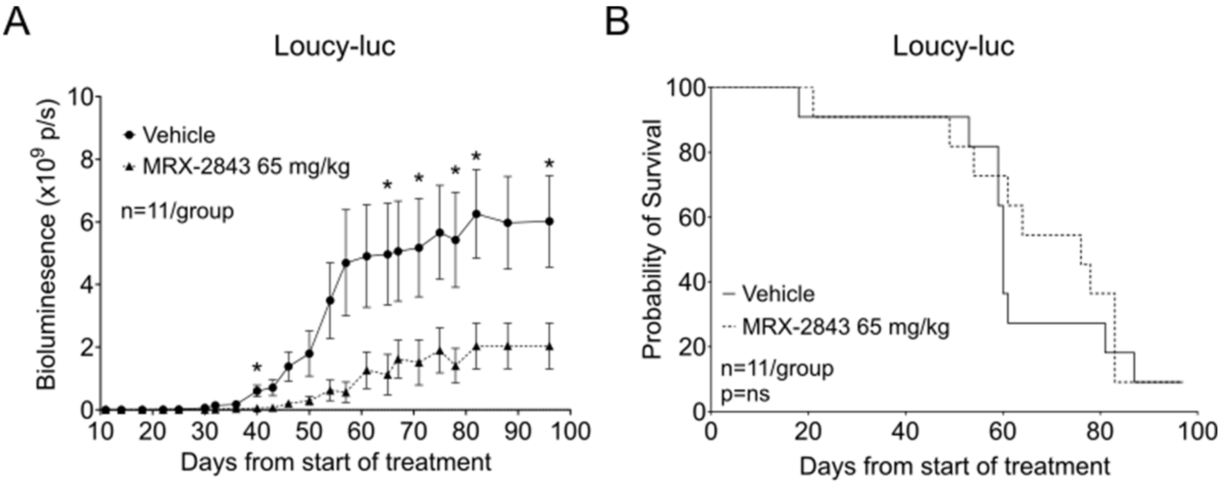

	Vehicle	MRX-2843 65 mg/kg
Median survival	60	76

Figure S2. MRX-2843 monotherapy delays disease progression and prolongs survival in an orthotopic ETP-ALL xenograft model. NSG mice were injected with luciferase-tagged Loucy cells and mice with established disease were treated once daily with 65 mg/kg MRX-2843 or saline vehicle (n=10/group). (A) Disease burden was monitored at intervals by bioluminescence imaging Mean values ± SEM are shown (*p<0.05, Mann-Whitney-U test). (B) Survival was monitored (p=ns log-rank test). Abbreviations: ns – not significant

Figure S3

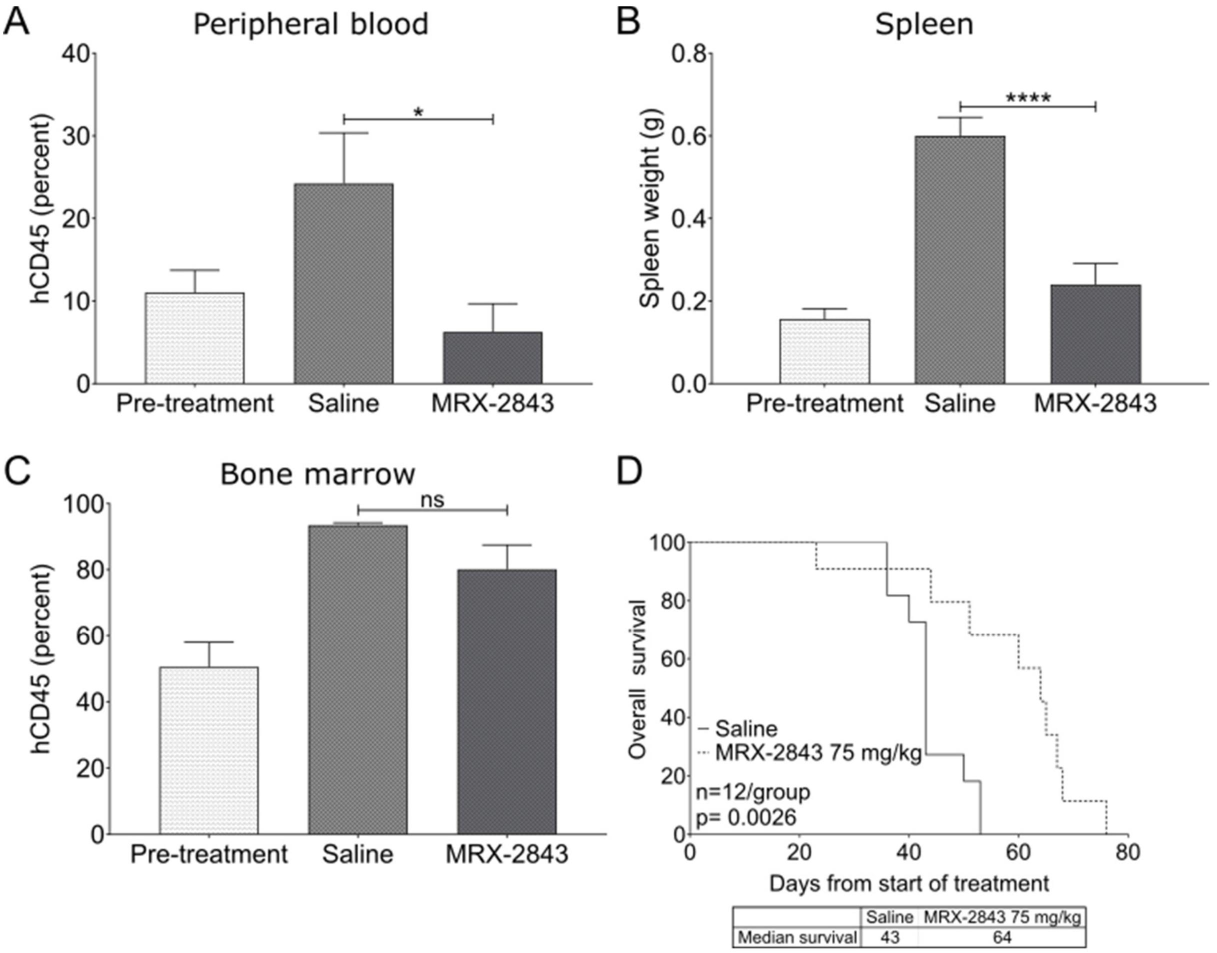

Figure S3. MRX-2843 monotherapy promotes leukemia clearance in the peripheral blood and spleen and prolongs survival in a patient-derived xenograft model of ETP-ALL. (A-D) NSGS mice were injected with mononuclear cells derived from a patient with ETP-ALL (ETP0068TJ) and treatment with 75 mg/kg MRX-2843 or an equivalent volume of saline vehicle was initiated 31 days later. (A-C) Peripheral blood, spleen and bone marrow were collected from mice (n=6/group) prior to the start of treatment (Pre-treatment) or after treatment for 29 days and leukemic blasts (hCD45+) were detected by flow cytometry. Spleen weight was used as a surrogate for splenic disease burden. Mean values ± SEM are shown (*p<0.05, ****p<0.0001, one-way ANOVA). (D) Mice were monitored for survival (p=0.0026, log-rank test, n=12). Abbreviations: ns – not significant
