# Supplementary material for: Therapeutic Targeting of MERTK and BCL-2 in T-Cell and Early T-Precursor Acute Lymphoblastic Leukemia
Source: Cancers (Basel). 2022 Dec 13;14(24):6142. doi: 10.3390/cancers14246142 (PMC9776749; doi:10.3390/cancers14246142)
Supplement: Supplementary file 1 [file cancers-14-06142-s001.zip › cancers-2019469-supplementary file 2.pdf]

Figure 1C

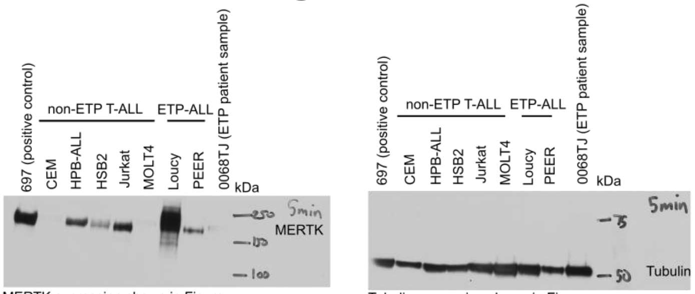

MERTK expression shown in Figure 1C. 5 minute exposure time

Tubulin espression shown in Figure 1C. 5 minute exposure time

Figure 1D

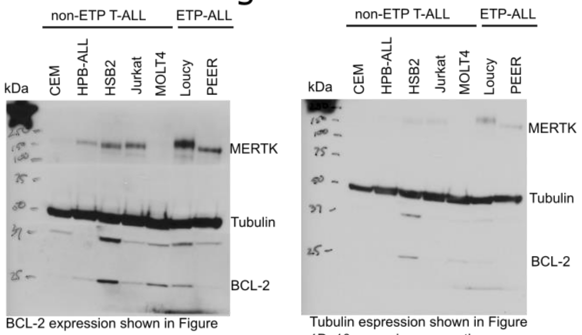

BCL-2 expression shown in Figure 1D. 2 minute exposure time

Tubulin espression shown in Figure 1D. 10 second exposure time

Figure 1E-F

Lanes labeled left to right

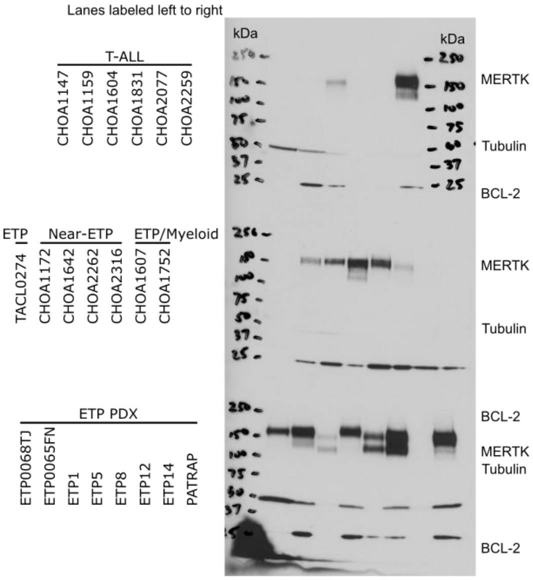

MERTK and BCL-2 expression shown in Figure 1E and 1F. 10 second exposure time. The tubulin antibody did not perform as expected, so the tubulin section of the blot was stripped an re-probed with fresh antibody and is shown separateley.

Lanes labeled left to right

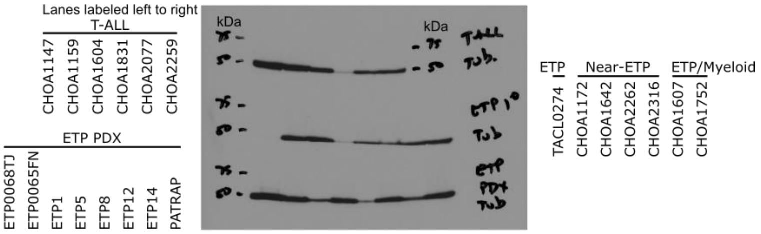

Tubulin expression shown in Figure 1E and 1F. 10 second exposure time.

Figure 2A

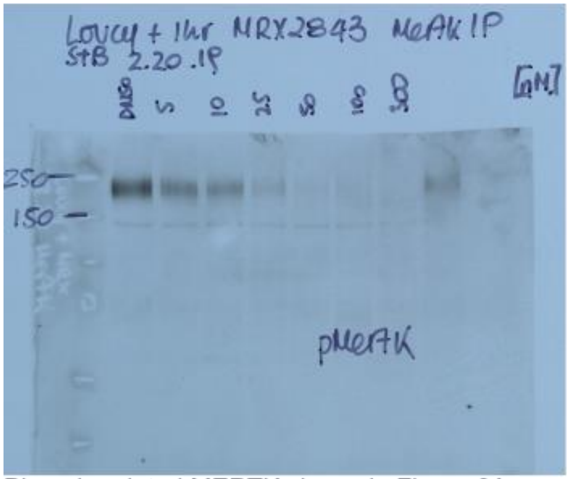

Phosphorylated MERTK shown in Figure 2A. This blot was also used for the quantitation shown in Figure 2C. Lane markers denote concentration of MRX-2843 in nM.

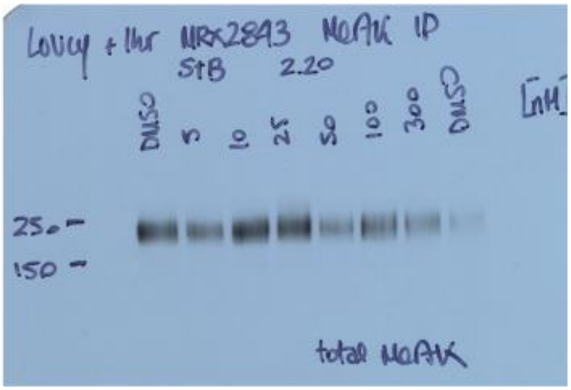

Total MERTK shown in Figure 2A. This blot was also used for the quantitation shown in Figure 2C. Lane markers denote concentration of MRX-2843 in nM.

Figure 2B

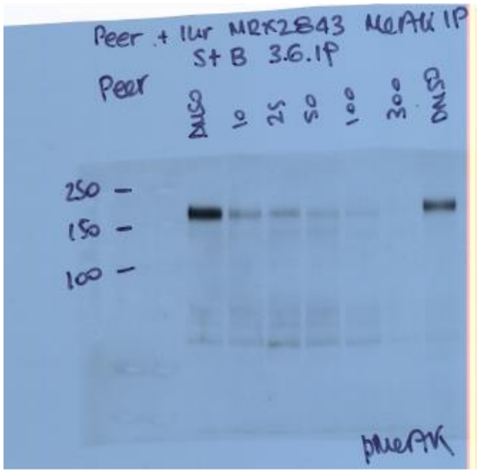

Phosphorylated MERTK shown in Figure 2B. This blot was also used for the quantitation shown in Figure 2D. Lane markers denote concentration of MRX-2843 in nM.

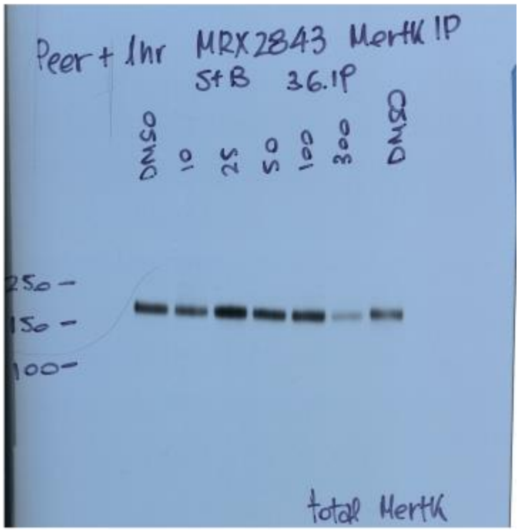

Total MERTK shown in Figure 2A. This blot was also used for the quantitation shown in Figure 2D. Lane markers denote concentration of MRX-2843 in nM.

Figure 2C

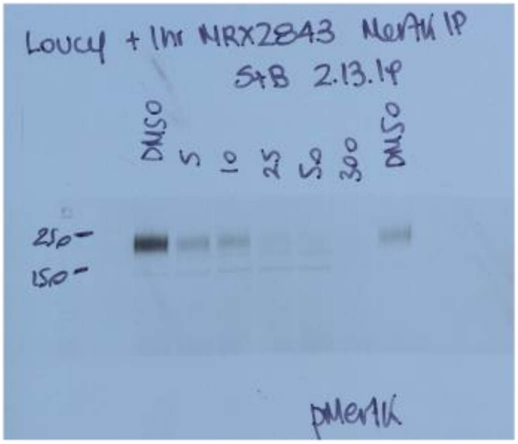

Phosphorylated MERTK blot used for the quantitation shown in Figure 2C. Lane markers denote concentration of MRX-2843 in nM.

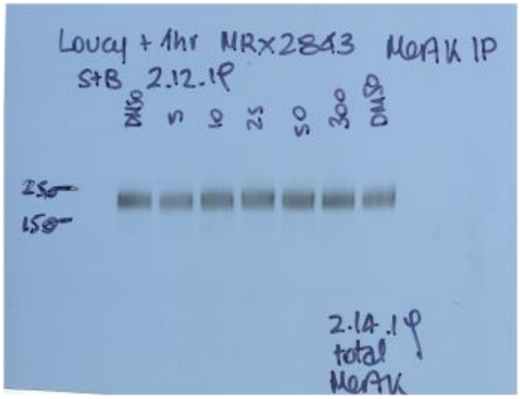

Total MERTK blot used for the quantitation shown in Figure 2C. Lane markers denote concentration of MRX-2843 in nM.

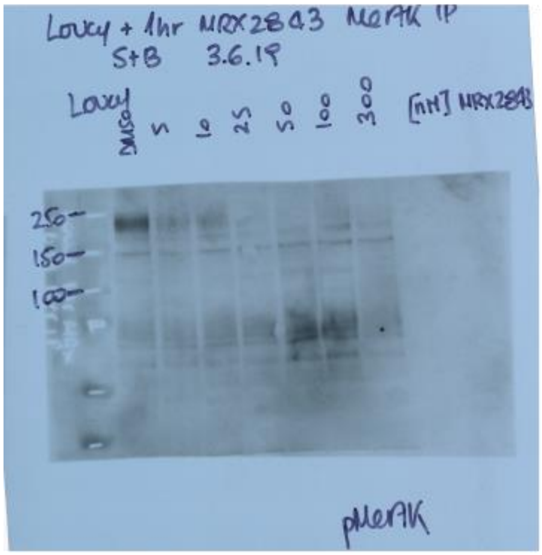

Phosphorylated MERTK blot used for the quantitation shown in Figure 2C. Lane markers denote concentration of MRX-2843 in nM.

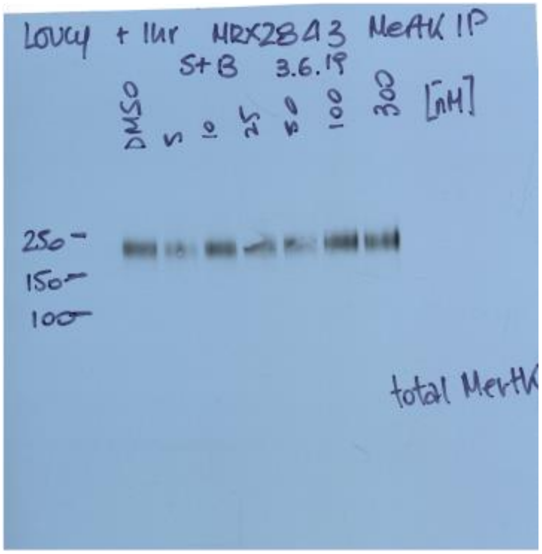

Total MERTK blot used for the quantitation shown in Figure 2C. Lane markers denote concentration of MRX-2843 in nM.

Figure 2D

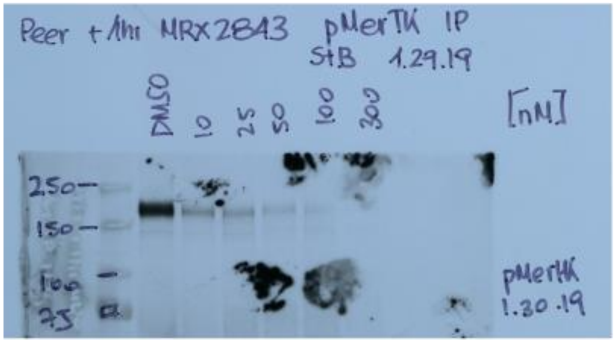

Phosphorylated MERTK blot used for the quantitation shown in Figure 2D. Lane markers denote concentration of MRX-2843 in nM.

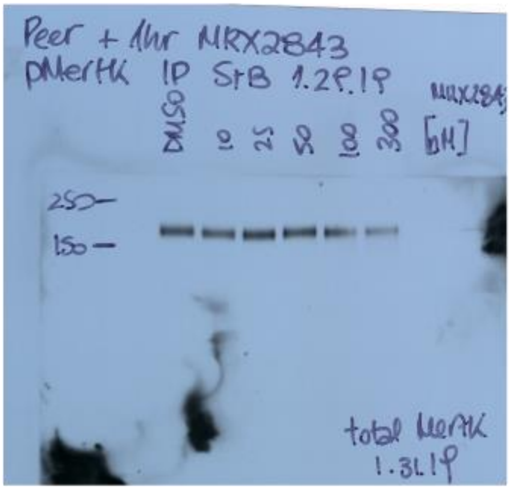

Total MERTK blot used for the quantitation shown in Figure 2D. Lane markers denote concentration of MRX-2843 in nM.

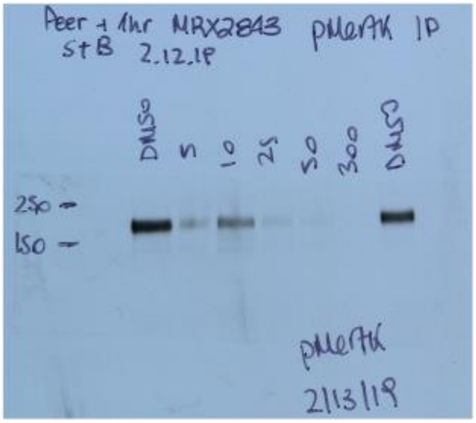

Phosphorylated MERTK blot used for the quantitation shown in Figure 2D. Lane markers denote concentration of MRX-2843 in nM.

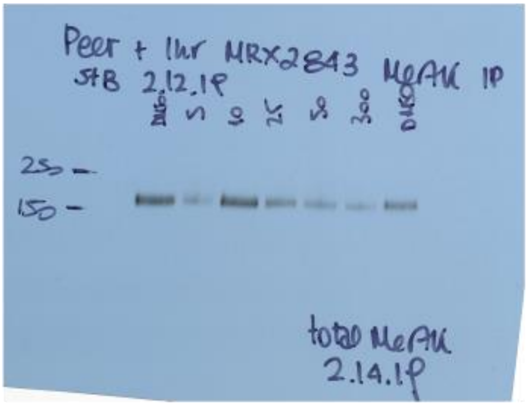

Total MERTK blot used for the quantitation shown in Figure 2D. Lane markers denote concentration of MRX-2843 in nM.

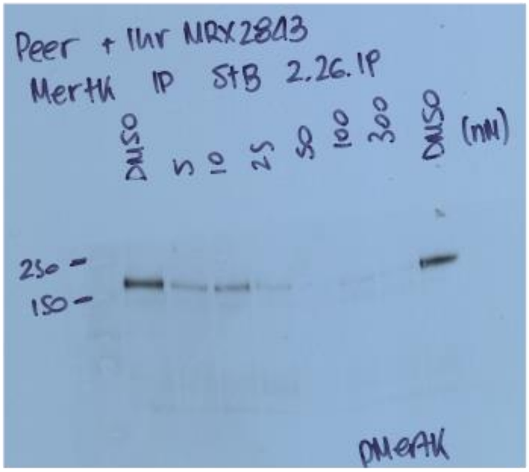

Phosphorylated MERTK blot used for the quantitation shown in Figure 2D. Lane markers denote concentration of MRX-2843 in nM.

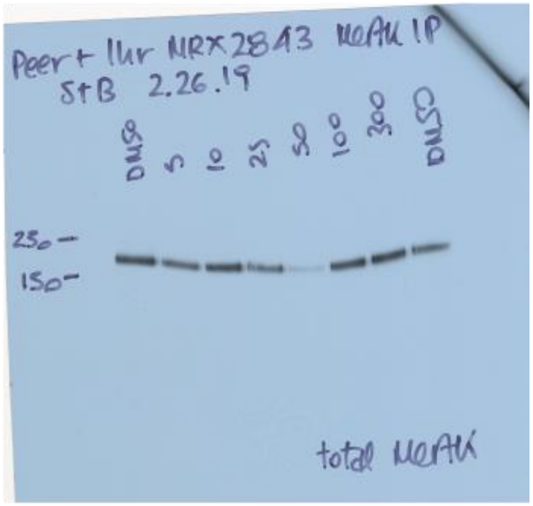

Total MERTK blot used for the quantitation shown in Figure 2D. Lane markers denote concentration of MRX-2843 in nM.

Figure 2E

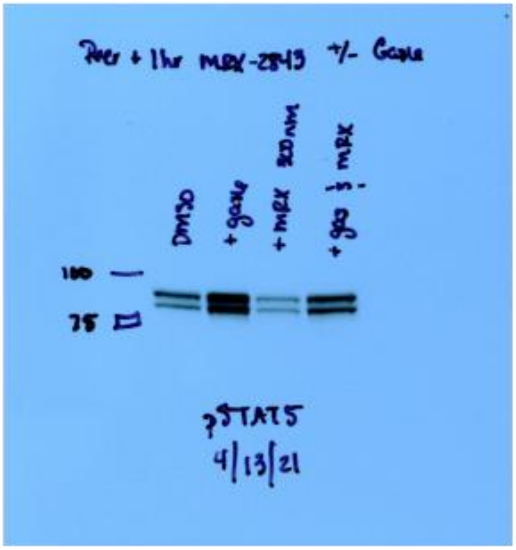

Phosphorylated STAT5 shown in Figure 2E. This blot was also used for the quantitation shown in Figure 2F. Lane markers denote presence/ absence of MRX-2843 and/or Gas6.

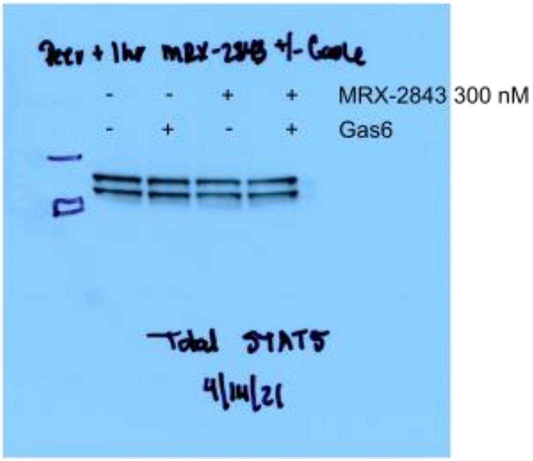

Total STAT5 shown in Figure 2E. This blot was also used for the quantitation shown in Figure 2F. Lane markers denote presence/absence of MRX-2843 and/or Gas6.

Figure 2F

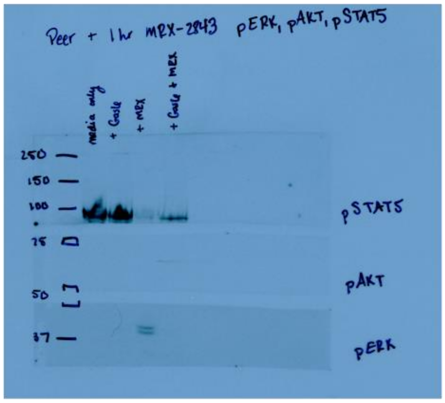

Phosphorylated STAT5 used for the quantitation shown in Figure 2F. Lane markers denote presence/absence of MRX-2843 and/or Gas6.

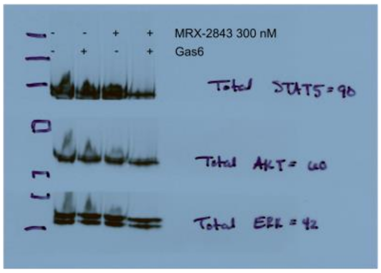

Total STAT5 used for the quantitation shown in Figure 2F. Lane markers denote presence/ absence of MRX-2843 and/or Gas6.

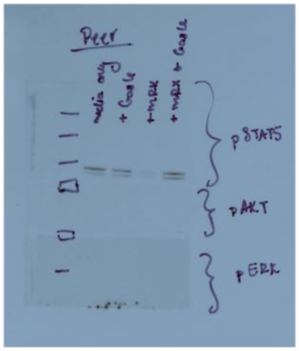

Phosphorylated STAT5 used for the quantitation shown in Figure 2F. Lane markers denote presence/absence of MRX-2843 and/or Gas6.

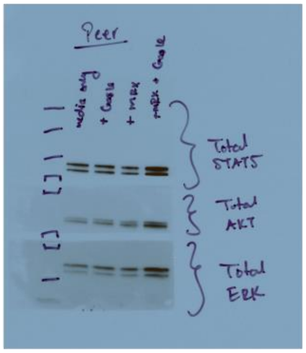

Total STAT5 used for the quantitation shown in Figure 2F. Lane markers denote presence/ absence of MRX-2843 and/or Gas6.

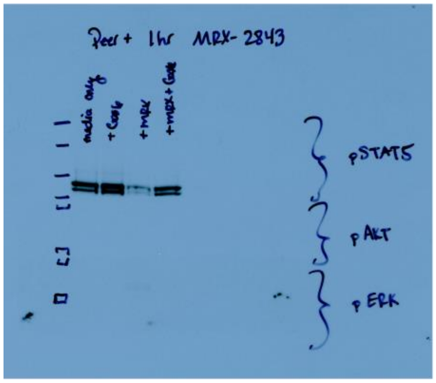

Phosphorylated STAT5 used for the quantitation shown in Figure 2F. Lane markers denote presence/absence of MRX-2843 and/or Gas6.

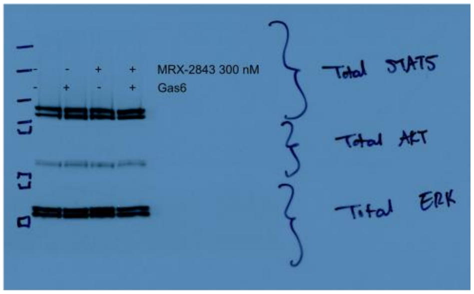

Total STAT5 used for the quantitation shown in Figure 2F. Lane markers denote presence/ absence of MRX-2843 and/or Gas6.

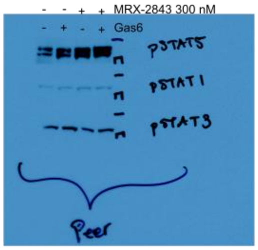

Phosphorylated STAT5 used for the quantitation shown in Figure 2F. Lane markers denote presence/absence of MRX-2843 and/or Gas6.

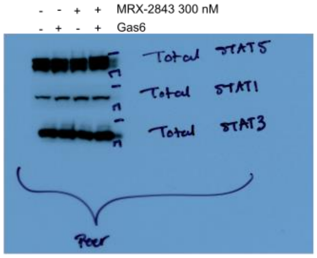

Total STAT5 used for the quantitation shown in Figure 2F. Lane markers denote presence/ absence of MRX-2843 and/or Gas6.
